# Magnetic resonance-guided focused ultrasound surgery for leiomyoma-associated infertility

**DOI:** 10.1016/j.fertnstert.2011.04.056

**Published:** 2011-05-13

**Authors:** Esther V. A. Bouwsma, Krzysztof R. Gorny, Gina K. Hesley, Jani R. Jensen, Lisa G Peterson, Elizabeth A. Stewart

**Affiliations:** a Department of Obstetrics and Gynecology, Division of Reproductive Endocrinology; b Department of Radiology, Mayo Clinic, Rochester, Minnesota

**Keywords:** Fertility, focused ultrasound, FUS, leiomyomas, MRgFUS, pregnancy, randomized clinical trial

## Abstract

**Objective:**

To describe magnetic resonance-guided focused ultrasound surgery (FUS) as a treatment for a case of leiomyoma-associated infertility.

**Design:**

Case report from a randomized clinical trial.

**Setting:**

Academic medical center.

**Patient(s):**

A 37-year-old woman with known leiomyomas and a history of 18 months of home-inseminations from a known donor.

**Intervention(s):**

Magnetic resonance-guided FUS treatment of uterine fibroids, where the dominant fibroid distorted the uterine cavity.

**Main Outcome Measure(s):**

Pregnancy.

**Result(s):**

A viable intrauterine pregnancy, with a full-term vaginal delivery, was conceived after a single clomiphene citrate and intrauterine insemination cycle.

**Conclusion(s):**

The role of FUS for enhancement of fertility in women with nonhysteroscopically resectable uterine fibroids distorting the uterine cavity should be investigated further.

Uterine leiomyomas are the most common tumors in women of reproductive age (1). The incidence of fibroids increases with age, and with the increased success of assisted reproductive technologies, more women of advanced maternal age are seeking pregnancies. Thus, the number of women seeking pregnancies and having leio-myomas is growing (2).

The relationship between leiomyomas and fertility has been the subject of debate for many years (3, 4). However, there is now general consensus that the location of the fibroid and its relationship to the uterine cavity are the key factors in whether the fibroid has a negative influence on fertility (5, 6). Submucosal fibroids are believed to decrease fertility rates the most, followed by intramural leiomyomas. Subserosal fibroids appear to have little to no effect on a woman's ability to conceive (5, 6).

For submucosal fibroids, it is agreed that myomectomy is beneficial and enhances the rates of fertility and live birth (5). However, for intramural fibroids there is not enough evidence to draw the same conclusions. It is not clear whether the difference in outcome is related to the biological differences between intramural and submucosal fibroids or to the fertility impairment from nonhysteroscopic surgeries for fibroids.

We report a case from a randomized clinical trial (NCT00730886, clinicaltrials.gov) evaluating fertility enhancement after magnetic resonance-guided focused ultrasound (FUS) or myomectomy in women with unexplained infertility, distortion of the uterine cavity, and nonhysteroscopically resectable uterine fibroids (type II submu-cosal and intramural). Focused ultrasound is a noninvasive therapy for treating uterine leiomyomas, and it was approved by the U.S. Food and Drug Administration (FDA) in 2004 for this indication. This technique converges multiple sound waves on a target site, causing localized coagulative necrosis, while guidance and thermal feedback are provided by magnetic-resonance imaging (MRI). Before conducting this trial, internal review board approval was obtained.

## Case Report

A 37-year-old Asian female in a committed same-sex relationship presented for an infertility consultation. She had been attempting to conceive witha known sperm donor and had been using home insemination timed by ovulation predictor kits for 18 months. She had one spontaneous miscarriage at 6 weeks with this approach. Three weeks before her consultation at our institution, she had been diagnosed with uterine leiomyomas. She also reported leiomyoma symptoms, including heavy, prolonged menses and intermenstrual spotting.

The patient underwent a basic infertility evaluation. An ultrasound examination showed several uterine fibroids and two hemorrhagic cysts in the right ovary, possibly consistent with endometriosis. A sonohysterography showed multiple fibroids with distortion of the uterine cavity, and an endometrial polyp was identified. Hysterosalpingogram showed normal opacification of both fallopian tubes and bilateral peritoneal spillage, and it confirmed the cavitary distortion. The ovaries appeared normal, with an antral follicle count of 18. Her cycle day-3 level of follicle-stimulating hormone (FSH) was 2.8 IU/L and of estradiol was 38 pg/mL. Her known sperm donor was a healthy 44-year-old male; his semen analysis was excellent, with a total motile sperm count of 141.8 million.

An MRI with gadolinium was acquired to further evaluate the fibroids, revealing an enlarged uterus measuring 6.2 × 9.4 × 9.1 cm. Multiple myomas were identified, with a dominant fibroid measuring 4.8 × 4.4 × 5.1 cm in the inferior right lateral myometrial wall abutting the endometrium. An intramural fundal fibroid measured 3.5 × 3.2 × 3.0 cm, and three other 1-cm intramural fibroids were identified. Again, small hemorrhagic ovarian cysts, possibly consistent with endometriosis, were seen.

**Figure 1:**
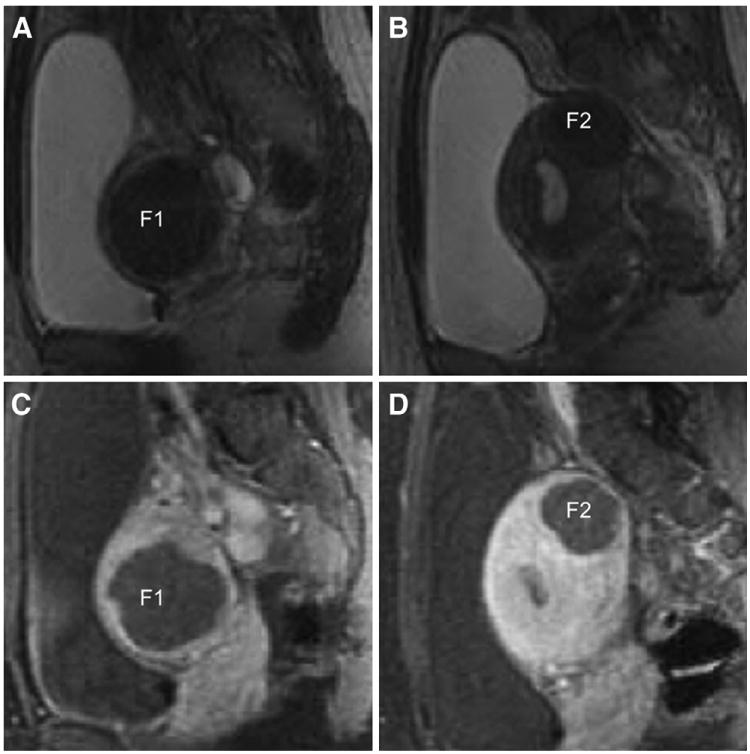
(**A–B**) Two fibroids (F1 and F2) on sagittal T2-weighted magnetic resonance imaging (MRI) before treatment. F1 is the dominant intramural fibroid in the right lateral myometrial wall abutting the endometrium. F2 is the smaller fundal fibroid. (**C–D**) Posttreatment sagittal T1-weighted images demonstrating a large area of nonenhancement in both fibroids after the administration of gadolinium. The nonenhanced areas correspond to nonperfused tissues resulting from focused ultrasound surgery (FUS) ablation. These images show the largest dimensions of the fibroids and do not illustrate the cavitary distortion.

The patient met the enrollment criteria for clinical trial NCT00730886; after we had obtained her written informed consent, she was randomized to the FUS arm. Treatment took place in two FUS sessions on two successive days without complications in August 2009. Only the dominant fibroid was treated in the first session, achieving a nonperfused volume (NPV) of 41%. In the second session, a NPV of 68% was achieved in the dominant fibroid, and four additional fibroids were ablated with similar results (Fig. 1).

During a subsequent visit in October 2009, a repeat sonohysterogram again showed a 7-mm polyp with no Doppler flow. A hysteroscopic polypectomy was performed, and the pathologic analysis confirmed a benign endometrial polyp.

In November, the patient took 50 mg of clomiphene citrate for 5 days starting from cycle day 3. On cycle day 14, she detected a luteinizing hormone (LH) surge by use of an ovulation predictor kit. Intrauterine insemination (IUI) was performed the next day with cryopreserved donor sperm (postwash sperm count of 20.8 million). A home pregnancy test was subsequently positive.

**Figure 2:**
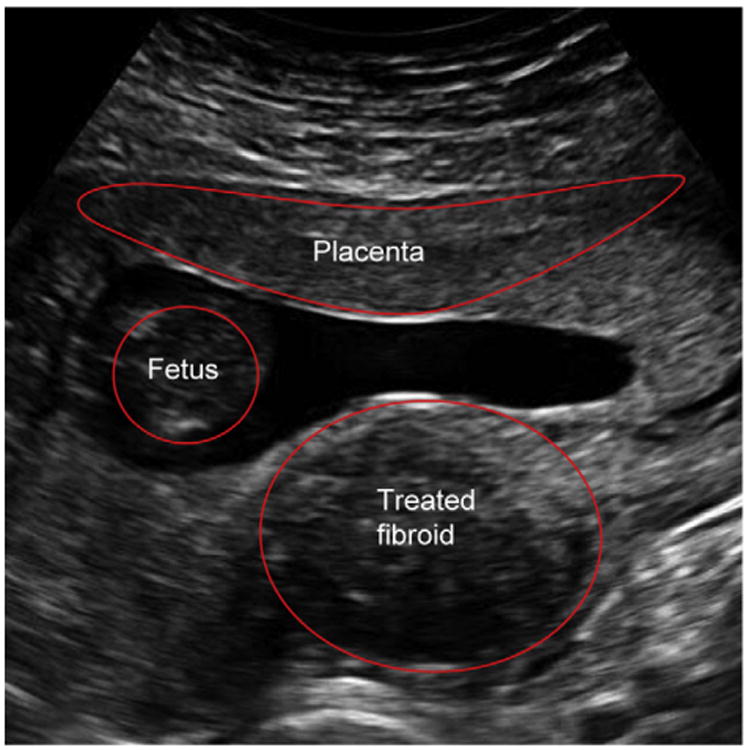
Transabdominal ultrasound image at 12 weeks' gestation showing the implantation of the placenta on the anterior wall. The treated dominant fibroid is located in the lateral myometrial wall and abuts the endometrial cavity.

The patient was closely observed during her pregnancy with frequent ultrasounds. The placenta implanted on the anterior wall with no relation to the treatment sites (Fig. 2). Fetal growth was normal, and the uterine fibroids remained stable in size. At 40 gestational weeks, the patient presented in labor with spontaneous rupture of membranes 24 hours before presentation. The patient had no fever and refused prophylactic antibiotics as recommended by her obstetric provider. After an uneventful labor, she gave birth to a 3,450 g girl, Apgar score of 7 and 9. Her postpartum course was complicated by a maternal temperature of 38.9°C, and she was started on intravenous antibiotics for endomyometritis; she was discharged afebrile 2 days later. The baby was treated with phototherapy and intravenous immunoglobulin for hyperbilirubinemia due to ABO incompatibility.

## Discussion

Reproductive dysfunction is a rare symptom attributable to leiomyomas (7). Recently, Pritts et al. (5) conducted an updated sys-tematic review of fibroids and fertility, and concluded that the clinical pregnancy rate of women with any type of fibroid is statistically significantly lower than in control women (RR 0.85; 95% CI, 0.73–0.98). However, in evaluating the effects on fertility by fibroid location, only submucosal and intramural fibroids have been found to have a negative influence on fertility. Moreover, for fibroids other than submucosal ones, surgical removal of the fibroids does not necessarily improve reproductive outcomes.

There are two ways of interpreting this discrepancy. First, the fertility-impairing characteristics of submucosal fibroids may be more profound than those of intramural fibroids. Karyotypes of fibroids differ by location within the uterus, which suggests that there may be biological differences (8). An alternative explanation is that morbidity from the myomectomy accounts for the difference. Thus, hysteroscopic myomectomy may provide a net benefit for fertility while laparoscopic or abdominal myomectomy and their increased risk of bleeding, adhesion formation, and infection may not (9, 10).

A patient with unexplained infertility, endometrial cavity distortion, and a nonhysteroscopically resectable fibroid is a clinical dilemma. Our trial was designed to address this question by randomizing women with unexplained infertility to myomectomy or FUS.

For patients who wish to conceive, FUS is a promising technique for treating leiomyomas. Since its introduction in 2004, there have been numerous reports documenting the safety and efficacy of FUS (11–18). Moreover, a recent case series reported on the safety of pregnancies after FUS (19). Fifty-four pregnancies in 51 women that occurred after FUS treatment were prospectively registered with the FDA. Live births occurred in 41% of the pregnancies, with an additional 21% ongoing beyond 20 weeks. There was a 64% vaginal delivery rate, and the mean birth weight was 3.3 kg, with no low birth weight infants. It is interesting that, in over a third of the patients who conceived after FUS treatment, infertility was a complaint prior to treatment (19).

In our patient, the distortion of the uterine cavity was not the only factor affecting her fertility. The patient also had undergone a hys-teroscopic procedure to remove a benign endometrial polyp. She elected to proceed with the clomiphene citrate plus intrauterine insemination treatment after that procedure, which most reviews suggest is an effective protocol for both unexplained and endometriosis-related infertility (20, 21). Unfortunately, it is impossible to tell which aspect of her treatment contributed to her enhanced fertility. Because of the limited recruitment of participants, this randomized clinical trial was closed by the sponsor in February 2010. Only one other patient participated in the study, and she was also randomized to FUS. After her treatment, she underwent two IVF cycles but has yet to conceive.

This case report shows that there may be a role for FUS for the enhancement of fertility in women with nonhysteroscopically re-sectable uterine fibroids and unexplained infertility. However, current experience is limited, and more systematic research is needed before any recommendations can be made.
